# Nanoparticles Enhanced Self-Driven Microfludic Biosensor

**DOI:** 10.3390/mi11040350

**Published:** 2020-03-27

**Authors:** Chunxiu Liu, Ning Xue, Haoyuan Cai, Jianhai Sun, Zhimei Qi, Peiyue Zhao, Fei Xiong, Zhaoxin Geng, Liying Jiang, Li Li

**Affiliations:** 1 State Key Laboratory of Transducer Technology, Aerospace Information Research Institute, Chinese Academy of Sciences, Beijing 100190, China; jhsun@mail.ie.ac.cn (J.S.); zhaopy@mail.ie.ac.cn (P.Z.); xiongfei198166@sohu.com (F.X.); 2 University of Chinese Academy of Sciences (UCAS), Beijing 100049, China; 3 School of Information Engineering, Minzu University of China, Beijing 100049, China; zxgeng@semi.ac.cn; 4 College of Electrical and Information Engineering, Zhengzhou University of Light Industry, Zhengzhou 450002, China; 5 College of Land Science and Technology, China Agricultural University, Beijing 100083, China

**Keywords:** microfluidic biosensor, amplification effect of nanoparticle, optomagnetic detection, sandwich assay

## Abstract

C-reactive protein (CRP) plays an important role in inflammation detection and disease monitoring. The optical biosensor is a highly sensitive and easy detection tool. The microfluidic self-driving optical sensors were fabricated with transparent glass material and used for the enhanced surface plasmon resonance (SPR) optical detection of the model protein CRP using Au nanoparticles (AuNPs) and a sandwich immune reaction. The 3D design of the chip was devised to improve the optical coupling efficiency and enable integration with a microfluidic control and rapid detection. The array of pre-fixed antibody modified by Au nanoparticles was used to achieve rapid antigen capture and improve the optical sensitivity. The Au nanoparticle amplification approach was introduced for the SPR detection of a target protein. CRP was used as a model target protein as part of a sandwich assay. The use of Au NP measurements to detect the target signal is a threefold improvement compared to single SPR detection methods.

## 1. Introduction

The optical bioassay methods are the most widely used techniques in all the detection approaches. The Surface Plasmon Resonance (SPR) technique has the advantages of a high sensitivity, wide application range, and real-time monitoring [1,2]. More methods are still being invested in to further improve SPR in terms of sensitivity and portability. The distinctive electronic and optical properties of the gold nanoparticle make it a research hotspot for signal amplification [1,2,3,4,5]. Optical bioanalysis is the most widely used technique.

Researchers used an effective sandwich immune method to link Au nanoparticles on labeled antibodies, so as to achieve the binding complex on the surface of the sensor sensitive membrane, to achieve the effective amplification of the signal, so as to an obtain ultra-high resolution, which can amplify the detection signal several or even dozens of times. Gold nanoparticles (AuNPs)-coupled SPR immunosensors have been explored to enhance the sensitivity of the detections system, and Au nanoparticles are recognized as SPR spectral sensitizers [1,6,7]. Gold and sulfhydryl (–SH) can combine to form a stable Au–S coordination bond, showing excellent performance for the binding and detection of antibodies and proteins [8,9]. It is considered that Au–Ab composite nanoparticles have more advantages than single nanoparticles in the high precision, high stability photo signal test. With the increase of the mass and refractive index of the composite nanoparticles, the magnification of the composite nanoparticles is larger than that of gold single nanoparticles. The nanotechnology plays a role in increasing the sensitivity of SPR for the detection of trace target molecules. The gold and magnetic nanoparticles, which can increase the sensitivity of SPR, were used as labels on antibodies (Ab), forming composite particles in sandwich assays, and the increase in size and weight of composite particles helped to amplify SPR signals for the detection of biomarkers at very low concentrations. Methods to detect protein biomarkers with a high sensitivity and ultralow detection limit (DL) have a potential value and application for the early diagnosis of diseases [9]. The monodispersed gold nanoparticles were used as labels to increase the sensitivity of the SPR imaging technique [10]. The amplification effect of antibody gold nanoparticles (AuNPs) and aptamer-AuNPs on the detection by SPR, and the development of a sandwich SPR method for protein detection, had been verified in previous research [2,3,11,12,13,14].

The microfluidic chip combined with total internal reflection imaging (TIRI) technology or SPR can be used as a point-of care testing (POCT) device for the detection of low concentration biomarkers [14,15,16,17,18]. A microfluidic chip was used to achieve the one-step detection of low concentration substances by integrating magnetic micro particle technology and the total reflection imaging technique [15]. The SPR (AuAg-SPR) sensors, based on gold-silver alloy film with wavelength interrogation, were fabricated to detect Cancer antigen 125 (CA125) by the sandwich immunoassay. The Au/Ag SPR sensors had a higher sensitivity than the conventional Au-SPR sensors in detecting CA125 [16]. An optics biosensor based on total internal reflection magnetic imaging (TIRMI) was fabricated for the point-of care testing (POCT) of the C-reaction protein (CRP) [17]. The microfluidic device was integrated with a surface plasmon resonance (SPR) sensor to realize a cost-effective multi-step and easy-to-use quantitative analysis. The SPR assay protocols with the device could be performed without any pumping system. To achieve portable applications without additional equipment, a self-driven microfluidic biosensor was designed, containing a plastic structure and various membranes with surface treatments to integrate with a SPR sensor for quantitative measurement [18,19]. 

This work developed the fast POCT detection device for low concentration CRP proteins via a self-driven microfluidic biosensor and modified by Au composite nanoparticles. The device could be used with the SPR, surface plasmon resonance imaging (SPRI), and TIRI during measurements.

## 2. Materials and Methods 

### 2.1. Chemicals and Apparatus

The human CRP antigen and anti-human CRP monoclonal antibodies (mAb) were purchased from Abzymo Biosciences Co. (Beijing, China). The capture mAb and labelled mAb are a pair of ligands. The labeled monoclonal antibody was coated with AuNPs. The Gold nanoparticles (AuNPs, 0.05 mg/mL, 10 nm diameter, pH = 7.7) were purchased from HyperCyte Biomedical Co. (Beijing, China). Phosphate buffer saline (PBS) buffer, 11-Mercaptoundecanoic acid, 1-(3-Dimethylaminopropyl)-3-ethylcarbodiimide hydrochloride (EDC), N-hydroxysuccinimide (NHS), Glutaric dialdehyde, sodium hydroxide (NaOH), 0.05 M Tris buffer, Tween-20, bovine serum albumin (BSA), and other reagents were obtained from Taidikang Biosciences Co. (Beijing, China). Poly(ethyleneimine) (PEI, m.w. 750,000), and polylysine (PLL) were purchased from Sigma-Aldrich Inc. (St. Louis, MO, USA). The buffer solutions were prepared with deionized water. The desired concentrations of reagents used in this study were prepared by serial dilutions in PBS. A HR2000 miniature optical fiber spectrometer (Ocean Optics, Florida, USA) was used for reagent detection, and a UV-2700 spectrophotometer (Shimadzu Company, Kyoto, Japan) was used for the comparative analysis of Ab-AuNPs and AuNPs. 

### 2.2. Structure of the Biosensor

The principle of the self-driving optical chip is a rapid sandwich immunoassay utilizing labeled Au particles in combination with optical signals system. The system innovatively combines the microfluidic chip, Au composite nanoparticles, and the optical module to achieve rapid detection of protein markers. 

**Figure 1:**
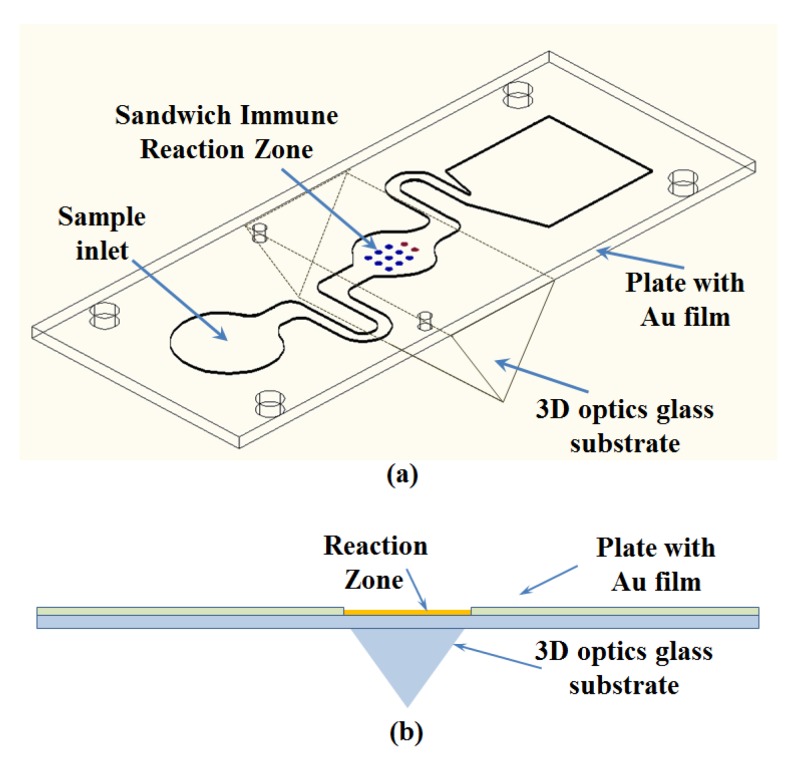
The design diagram of the microfluidic chip: (**a**) 3D diagram of the chip, and (**b**) front view of the chip.

The assay platform in this work is a microfluidic chip system. Each chip contains 3D optical coupled cavities and a microfluidic channel composed of an inlet chamber, a reaction tank, and a waste liquid reservoir (Figure 1a,b). The chip was fabricated from a glass layer and Polymethylmethacrylate (PMMA) bonded by a pressure sensitive adhesive (PSA). The basic structure of the double-sided adhesive was pasted on the surface of the 3D glass base plate. The capture mAb and labelled mAb were the reactions in sequence on the bottom of the reaction chamber. The self-driving was realized by capillary action and the PSA hydrophilic membrane. There is a pinhole outlet and suction pad to avoid the possible spillage of the infection. 

### 2.3. Modification Method and Amplification and Principle of the Biosensor

**Figure 2:**
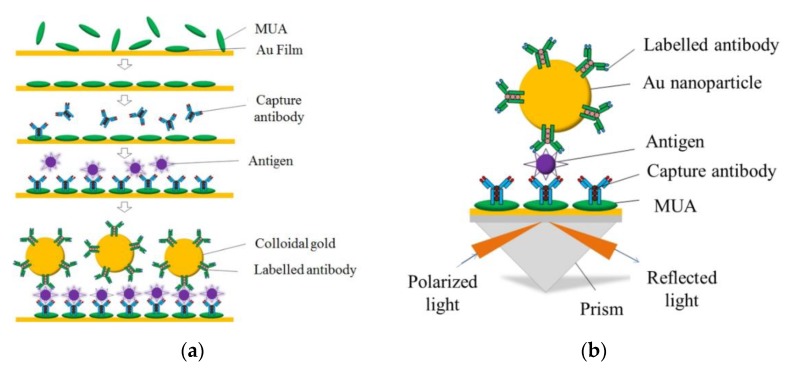
(**a**) Schematic of the oriented immobilization of the AuNPs-antibody onto the assembled SPR sandwich immunosensor chip. (**b**) Schematic of the SPR sandwich immunosensor chip with the AuNPs-monoantibody bioconjugate for signal amplification.

First, the device slices covered by the Au film were previously immersed in 11-Mercaptoundecanoic acid (150 mM) ethanol solution for 24 h at room temperature to form the thiols self-assembled monolayer (SAM) in fume hood (Figure 2a). Then, the devices were sequentially washed thoroughly with deionized water. After that, 0.2 mol/mL EDC solution and 0.05 mol/mL NHS solution were flowed for 50 min to ensure that the carboxyl groups (COOH) were activated to the covalently immobilized CRP capture antibody (50 μg/mL). According to the different detection purposes, the CRP capture antibody can be fixed on the sensor surface evenly or fixed by a pL-sized arrayed dispensed point sample. In order to inhibit the non-specific adsorption and deactivate the unreacted NHS ester groups on the surface, BSA solution (10 mg/mL) was added and incubated for 1 h. Then, the CRP solutions (0.5–500 ng/ mL) were injected into the chamber in sequence for specific binding with the capture antibody, and each concentration gradient of the CRP solutions was observed for 30 min. Finally, the CRP labeled antibody (50 μg/mL) was applied to form a sandwich reaction, further enhancing the SPR signal. The Au nanoparticles labeled antibody, instead of the labeled antibody, was added for the enhanced contrast test.

The three-dimensional dynamic plasma field of the sandwich can be enhanced by the coupling of the nano-composites and the plasma. This field overlaps with the target molecule, interacts with the others, produces a local light field which can resist the interference of a specific signal, and realizes the rapid enrichment of ultra-high power signals and optical nonlinearity. In this study, the CRP mode protein was used for detection, and the spectral shift signals caused by the labeled antibody and the labeled antibody AuNPs were compared and analyzed to verify the field enhancement effect [16,18].

The measurement principle of the 3D chip is a rapid sandwich immunoassay labeled by Au nanoparticles, as shown in Figure 2b. A 10 µL volume of the buffer standard sample was loaded into the chip. The CRP antigen reacted with the immobilized capture mAb, and the complex was trapped on the chip interface, after which the labelled mAb-Au complex was added and the sandwich immune reaction took place. Finally, the capture mAb-CRP-labelled MAb-Au nanoparticles complex gradually formed and was detected by SPR. The optical signals were a logarithmic correlation to the CRP antigen concentrations. The sample was collected in a waste tank within the chip and then disposed of after the detection.

The tunable optical properties of metal nanoparticles give a particular role to SPR signal enhancement [20,21,22,23]. The special optical properties of the gold nanostructures cause the involvement of the particle’s free electrons in the localized surface plasmon (LSP). The electrical fields of the particle’s surface are increased, and the particle’s optical extinction reaches the maximum at the plasmon resonance frequency [24,25,26,27,28,29]. Au nanoparticles are the most widely used ones as they are chemically stable and resistant to surface oxidation.

## 3. Results

### 3.1. Modification of the 3D Biosensor

#### 3.1.1. Preparation of Antibody-coated Gold Nanoparticles and Spectral Analysis and Comparison of Ab-AuNPs

0.1 mL of paired monoclonal antibody solution (100 mg·L^−1^ at 5 mm PBS, pH = 7.4) was added to 0.9 mL of AuNPs buffer solution and mixed gently by pipette, and incubated at room temperature for 30 min to form antibody-coated AuNPs (Ab-AuNPs). Due to the reaction of the SH group of protein with gold, the AuNP antibody complex was formed upon the formation of the gold sulfur bond. We added 0.2 mL BSA solution (10% (w/v) in 50 mm PBS, pH = 9.0) to block the reaction, incubated it at room temperature for 30 min, labelled it Ab2 -AuNPs, centrifuged it at 10,000 × g for 10 min, and then suspended the particles in PBS buffer. The surface of the chip was fully rinsed with PBS after each step.

**Figure 3:**
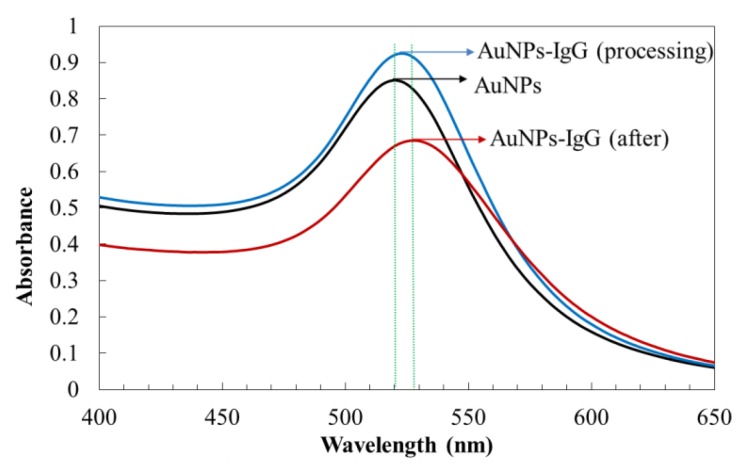
The UV-Vis absorption spectra comparison of gold nanoparticles and AuNPs-IgG complex particles.

The UV-Vis absorption spectra of AuNPs and AuNPs-IgG are shown in Figure 3. As shown in the figure, the surface plasmon resonance absorption peak of AuNPs was 520 nm with a symmetrical peak shape and narrow half-peak width, which indicated that the prepared AuNPs had a good dispersion and spherical structure. After adding the IgG labelled antibody protein, the maximum absorption occurred. The peak redshift, peak intensity, and half-peak width increased, which further indicated that AuNPs formed new complexes with antibody proteins. The absorption peak of AuNPs-IgG was 530 nm when the binding reaction was complete. The AuNPs-IgG were centrifuged and re-suspended in PBS-buffer. 

### 3.2. The CRP Detection with the 3D Biosensor

#### 3.2.1. Sensor Response

**Figure 4:**
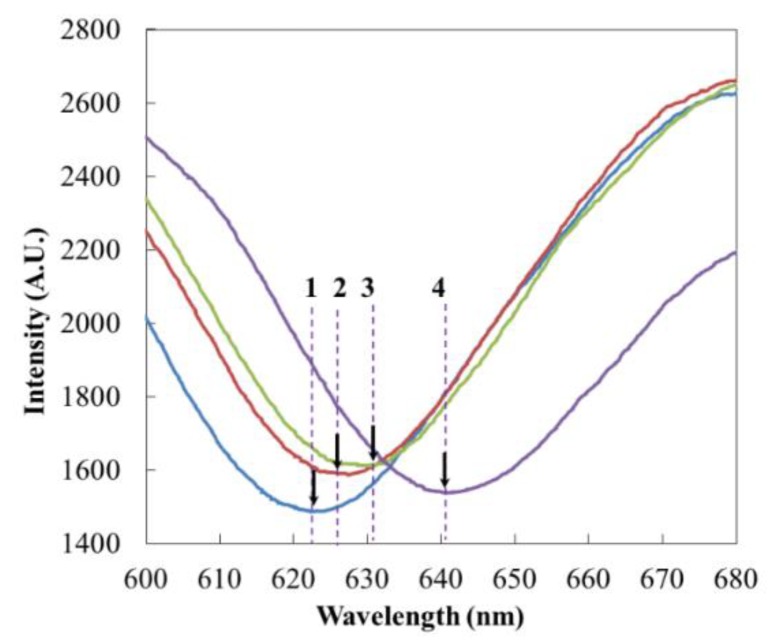
Au NP-amplified immunoassays and the corresponding SPR signals. 1: PBS, 2: Capture antibody, 3: antigen (500 ng/mL CRP), and 4: labelled Ab-AuNPs.

The PBS buffer, capture Ab, Ag and labelled Ab were sequentially added to the reaction chamber, and the peak value gradually produced a red shift (Figure 4). When the reagent was added, a red shift occurred instantaneously; then, with the reaction proceeding, the red shift gradually proceeded, reaching stability at about 30 min. After each step of the reaction, PBS rinse was used to remove unreacted substances. Within 30 min of capturing the antibody binding, the absorption peak changed from 623.10 nm to 625.80 nm, shifting 2.70 nm to the right. The PBS phosphate buffer did not change after cleaning, which indicated that the binding of the antibody was relatively firm. The highly active EDC/NHS solution prepared before the experiment had a good activation effect on the carboxyl groups in the SAM layer.

#### 3.2.2. CRP Detection

**Figure 5:**
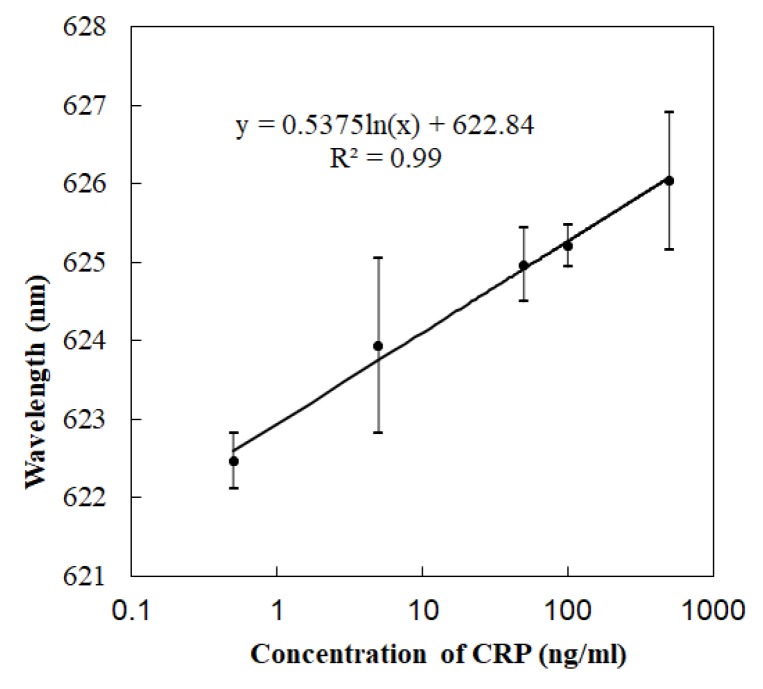
The SPR immunosensor results for the CRP standard buffer samples (0.5–500 ng/mL) assayed using Au nano-particle labels. Error bar with 3 tests.

According to the concentration gradient (0.5 ng/mL, 5 ng/mL, 50 ng/mL, 100 ng/mL, and 500 ng/mL), the CRP antigen solution was added, and the data were recorded (Figure 5). The concentration gradient reaction is not a linear reaction. According to the experimental data, the absorption peak shifted right to 627.82 m and changed 4.10 nm when 500 ng/mL CRP was added. With the addition of labelled Ab, it moved right and increased by 2.71 nm. In the contrast experiments, the absorption peak shifted 8 nm with the addition of the labelled antibody-AuNPs composite particles. In the non-specific adsorption contrast experiments, there was no obvious red shift of the absorption peak after PBS washing after gold particles were added. The detection limit of this study changed for different modified states. In this experiment, the lowest concentration of 0.5 ng/mL was used to show that it had an obvious spectral shift. Since the optical detection of SPR was not a linear concentration reaction, the logarithmic data processing was carried out. The data of three tests were sorted out via the error bar. The repeatability and consistency of detection needs to be improved in future research.

Table 1 is a set of comparative test data. The peak red shift data of the capture Ab and 50 ng/mL CRP in the two groups were basically the same, while the peak red shift of the labelled antibody-AuNPs composite particles was significantly higher than that of the labelled antibody, indicating that gold nanoparticles played an obvious role in the amplification reaction.

## 4. Discussion

The sensitive quantitative detection of protein biomarkers is very important for the diagnoses of various diseases and the monitoring of their treatment. The low sensitivity of conventional SPR methods cannot achieve an adequate assessment of the disease and the timely and dynamic effectiveness of the treatment. These limitations apply to many of the assays for human inflammation biomarkers (CRP, PCT, interleukin 6). In this work, we designed and developed a sensitive method for the detection of CRP using a sandwich immunoassay with an AuNP-enhanced SPR chip for the early diagnostics of inflammation.

The integration of nanoparticles and microfluidic chips is helpful for the automation of sample processing, mixing, and incubation to achieve one-step detection, and plays an important role in improving the reliability and repeatability of the determination and the user-friendliness of the technology. The low-cost microfluidic platform for the detection of proteins ensures fast and reliable results due to a minimum of manual steps involved. 

Reasonable chip design and modification is the key point for SPR sensor to achieve sensitive response and rapid detection. The design and preparation of micro flow tank and micro reaction chamber should take into account the automatic and smooth sample injection and effective sample reaction space. Therefore, high-precision micromachining and nano modification processes are helpful for chip preparation, accurate sample size control and automatic sample injection.

A self-driving microfluidic chip was designed to achieve fast detection by integrating three-dimensional chip fabrication and Au composite nanoparticles. The use of AuNP measurements to detect target signals is a threefold improvement when compared to direct sandwich SPR detection methods. The optical assay of standard CRP was studied in order to develop applicable 3D devices and light enhancement methods for precise and fast protein markers detection at low concentrations with a small sample at the microlitre level. As a new application, the combination of gold particles with 3D sensors lays a foundation for POCT.

The developed technology can be used for the analysis of other low-concentration protein biomarkers. Furthermore, this technique can be applied to analyse multiple biomarkers simultaneously in one sample of blood serum using several combination of antibody reagents, followed by a synchronized spot optical imaging detection using a section-fixed spot of the chip with the corresponding immobilized capture Ab. In future work, we will extend our current work to multiple biomarkers via the extension of the chip system, with no need for advanced sample preparation and user intervention.

## Figures and Tables

**Table 1:** Specificity analysis of the fabricated SPR biosensor.

Specificity Analysis	Spectral Shift (nm)		
	Capture Antibody	Antigen	Labeled Antibody
Sandwich immunoassay	2.70	3.58	2.70
AuNPs-enhanced immunoassay	2.69	3.14	8.96
